# Social determinants of health screening tool: systematic review and Delphi study

**DOI:** 10.3399/BJGPO.2024.0274

**Published:** 2025-12-19

**Authors:** Emma Parry, Ross Wilkie, Kate Warren

**Affiliations:** 1 Primary Care Centre Versus Arthritis,School of Medicine, Keele University, Staffordshire, UK; 2 Population Health Unit, The Royal Wolverhampton NHS Trust, Wolverhampton, UK

**Keywords:** social determinants of health, health inequities, primary health care

## Abstract

**Background:**

Social determinants of health (SDOH) critically influence population and individual-level outcomes, but we do not collect this information routinely in primary care.

**Aim:**

To develop a screening tool for SDOH relevant to UK settings using systematic review and Delphi methodology to identify suitable questions.

**Design & setting:**

A systematic review and Delphi study were undertaken.

**Method:**

For the systematic review, five databases and grey literature were searched. Selected studies included questions or tools that screened for SDOH relevant to UK settings. Included questions and tools were measured against the eight gold standard steps for measure development. Data were thematically analysed and arranged into pre-specified domains. For the Delphi study, individuals with an interest in SDOH were invited to take part in a three-stage modified Delphi study. Ranking of 172 items in survey 1, rating of 111 items in survey 2, and ranking of 56 items in survey 3 led to one question being selected per 10 pre-specified domains. Inductive content analysis of free-text responses from the surveys was performed.

**Results:**

Of 7889 citations, 104 studies were included in the systematic review. Screening primarily took place in clinical settings using written formats. Seven participants took part in the first Delphi survey. Prioritised questions were direct, had binary answers, had specific wording, were concerned with current situation, and had immediate impacts on health.

**Conclusion:**

The review provides a comprehensive overview of screening questions and tools for collecting information on SDOH. We present a 10-item screening tool from the highest ranked questions that can be used to screen for SDOH in primary care settings in the UK.

## How this fits in

Reliance on area-level data to inform resource allocation, policy, and epidemiology on deprivation is problematic. Gathering individual-level data on people’s social determinants of health (SDOH) in primary care could improve health outcomes, ensure resources are directed where they are needed most, and improve population-level data. Our synthesis of questions and tools, collecting information on people’s SDOH led to the development of a UK-specific SDOH screening tool through a Delphi consensus process. This tool could improve direct patient care, identify people with unmet social need, and support accurate service planning.

## Introduction

Social determinants of health (SDOH) are the non-medical factors affecting health. These factors are the conditions in which people are born, grow, work, live, age, and the wider set of forces and systems shaping daily life (for example, someone’s living conditions and ability to buy food).^1^ SDOH critically influence up to 80% of health outcomes,^2^ yet individual-level social risks are not collected routinely or in a standardised format in UK healthcare settings. People from more deprived backgrounds face higher mortality rates and worse ill health.^3^ Indices of Multiple Deprivation are the routinely used measure in England for deprivation based on geographical area,^4^ typically containing an average of 672 households.^5^ This information is used in research, clinical evaluation, and in clinical decision making (for example, risk stratification tools such as QRISK).^5^ Using area-level measures to target resources to individuals, however, is problematic as we cannot assume that individual and area-level information is interchangeable.^6^ This is especially the case in rural populations with higher variability in deprivation.^7^

The influence of SDOH on primary care consultations is unavoidable^8^ and there is evidence that having better information on individual-level social risks and needs can improve health outcomes during consultations through adaptations of care plans and being mindful of financial barriers to health care.^9,10^ Identifying social risk factors has been shown to benefit individuals through increased referral to community services and resources, reduced hospitalisations, improved chronic disease management, and positively impacting on mental health.^11^ Collecting and recording individual SDOH will aid research on health inequalities, prioritising understanding the implications of SDOH on disease outcomes and incidence rates contributing to public health policy.^12^

The NHS is committed to addressing health inequalities and recognises the need for better data. Individual-level SDOH data are particularly lacking, but the primary electronic health records (EHRs) may have a unique role. A systematic review identified largely US-based multi-domain social risk tools, that exist for collecting information on SDOH EHRs^13^ but key uncertainties remain regarding applicability in the UK.

Our study aims to update this review and synthesise questions and tools for gathering relevant SDOH information relevant to UK settings, assess adherence to gold standard tool development measures, and gather insights on screening practicalities through a systematic review. Second, we sought consensus using Delphi methodology on what questions should be included in a SDOH screening tool for use in UK primary care.

## Method

### Part 1: Systematic review

The reporting of the systematic review was guided by Preferred Reporting Items for Systematic review and Meta-Analyses (PRISMA) guidelines.^14^

#### Information sources

We searched English language-only literature using MEDLINE, CINAHL, Embase, Web of Science, Social Science and Practice databases from October 2002–October 2022. Government websites and the first 20 pages of Google Scholar were used to search for grey literature using the search terms: 'social determinants of health' and 'screening' (Supplement 1).

Previous systematic reviews involving social determinants of health were used for reference checking after the full-text stage,^15,16^ in addition to a US-based screening tool comparison table.^17^

#### Eligibility criteria

The eligibility criteria were published manuscripts that included questions that fitted into the pre-specified domains as determined by PROGRESS PLUS, which is endorsed by the World Health Organization (WHO):^1,18^ finance, housing or homelessness, food security, transport, utilities, education, neighbourhood safety, social connectedness, childcare, and employment.

#### Selection process

One author (EP) screened titles and abstracts and assessed full texts for inclusion against eligibility criteria (Table 1) with 10% randomly allocated to a second reviewer (KW) for independent review. Disputes were resolved by third reviewer arbitration. Further studies were identified through citation checking of included full texts.

**Table 1.** Eligibility criteria

*Inclusion*	*Questions concerning adults* *Questions or tools collecting information on social determinants of health in published manuscripts* *Full question and answers presented* *Questions relevant to UK settings* *Questions asking details up to 12 months previously*
*Exclusion*	*Full text not available* *Non-English language* *Questions including actual income* *Abstracts* *Tools requiring licences or payment to access and use*

#### Data collection process and data items

Data were extracted by EP into a piloted Excel spreadsheet. We extracted data on year of publication, participant age, country, sample size, study design, population screened, screening setting, screening format, administration method, duration of screening, name of tool or source of questions, how questions were developed, information on validity and feasibility of questions, the question(s) and answers.

#### Synthesis methods

First, questions were arranged into their corresponding domains using categorical content analysis methods.^19^ Second, the questions were analysed thematically,^20^ which involved identifying themes and interpreting patterns leading to the development of subheadings. Questions and tools were split into those suitable for screening (that is, short and no more than 1–2 items long) and those suitable for more in-depth questioning (that is, longer tools or questions with ≥3 items). The final themes were reviewed by our patient and public involvement and engagement (PPIE) group.

#### Risk of bias assessment

A formal risk of bias assessment of the included studies was not conducted, however, we did assess the extent each reported tool or question, met the eight gold standard steps of measure development (Supplementary Table S1).^13^ These steps included the following: (1) reporting of construct definition; (2) items generated by experts; (3) testing initial items on a representative sample; (4) testing validity and reliability tests on a pilot; (5) tool refined based on the pilot; (6) re-testing refined instrument; (7) retest validity and reliability; and 8) reporting of psychometric properties.^13^

#### Patient and public involvement and engagement

A PPIE group were involved in the design of this study, agreeing questions for inclusion and reviewing the themes generated from the data synthesis.

### Part 2: Delphi study

Reporting of the Delphi study was guided by the Guidance on Conducting and REporting DEiphi Studies (CREDES) checklist (Supplement 2).^21^

#### Design

A three-step modified Delphi method was conducted between November 2023 and May 2024, with three rounds of online surveys administered using LimeSurvey. Items included in the first survey were informed by findings of the systematic review.^22^ Each online survey was open for response for 6 weeks, and two reminders were sent to those with incomplete or no responses.

#### Panel selection

The panel were recruited through social media, local hospital, and integrated care board newsletters and emailing local professional networks. We purposively invited experts working in community settings along with public and patient members to ensure the panel consisted of individuals from different professional backgrounds with experience working with people from underserved communities. For survey 1, there were 27 participants, which comprised: GPs (*n* = 8), PPIE members (*n* = 7), third-sector workers (*n* = 3), public health specialists (*n* = 2), midwives (*n* = 2), social worker (*n* = 1), district nurse (*n* = 1), advanced nurse practitioner (*n* = 1), healthcare assistant, (*n* = 1), and local authority worker (*n* = 1) (Supplementary Table S2).

#### Round 1

The survey was organised into the 10 domains pre-specified for the systematic review: transportation, utilities, food security, housing or homelessness, financial strain, social connections, childcare, employment, education, and neighbourhood safety. Only screening questions that met at least one of the gold standards checklist were included in the Delphi study (Supplementary Table S1). Participants were asked to rank questions in each domain with the top-ranked question being the item they felt captured the best information on social risk related to that domain. Across the 10 domains 172 questions were ranked in total.

Collective mean, median, mode, standard deviation (SD), interquartile range (IQR), variance and rank, according to mean ranking score, were calculated for each domain. A top-half ranking method was used and only items that ranked in the top half were taken through to the next round.^23^

#### Round 2

Delphi participants were sent feedback summarising results from round 1. In round 2 the survey was reorganised into 11 domains to improve clarity, adding in living conditions. Participants were asked to rate 111 questions, on a five-point Likert scale from extremely important to not at all important thinking about which question is most important for understanding the corresponding domain in a screening tool. For each item mean, median, mode, and percentage agreement with extremely important and very important were calculated. Those items with 67% agreement or above were retained for survey 3.^24,25^ Responders were also asked to rank the domains by order of importance.

#### Round 3

Participants were asked to rank 56 questions with the top-ranked question per domain being the item that was felt most appropriate to understanding access to or situation with regard to social risk. Responders were again asked to rank the domains by order of importance.

Spearman’s rank correlation coefficient was used to determine correlation with ranking of domains by order of importance between survey 2 and 3.

Note no modification of items was permitted between rounds as this would have altered the validity and reliability of items.

#### Free-text analysis

During each round, within each domain, panel members were provided with a free-text box to either comment on the questions presented to them or to give reasons behind their responses. The free-text feedback comments were analysed using inductive content analysis.^26,27^

#### Patient and public involvement

Our PPIE group piloted survey 1, advised on clarification of certain terms and layout.

#### Statistical analysis

Data were analysed with Microsoft Excel 2404.

## Results

### Part 1: Systematic review

**Figure 1.**
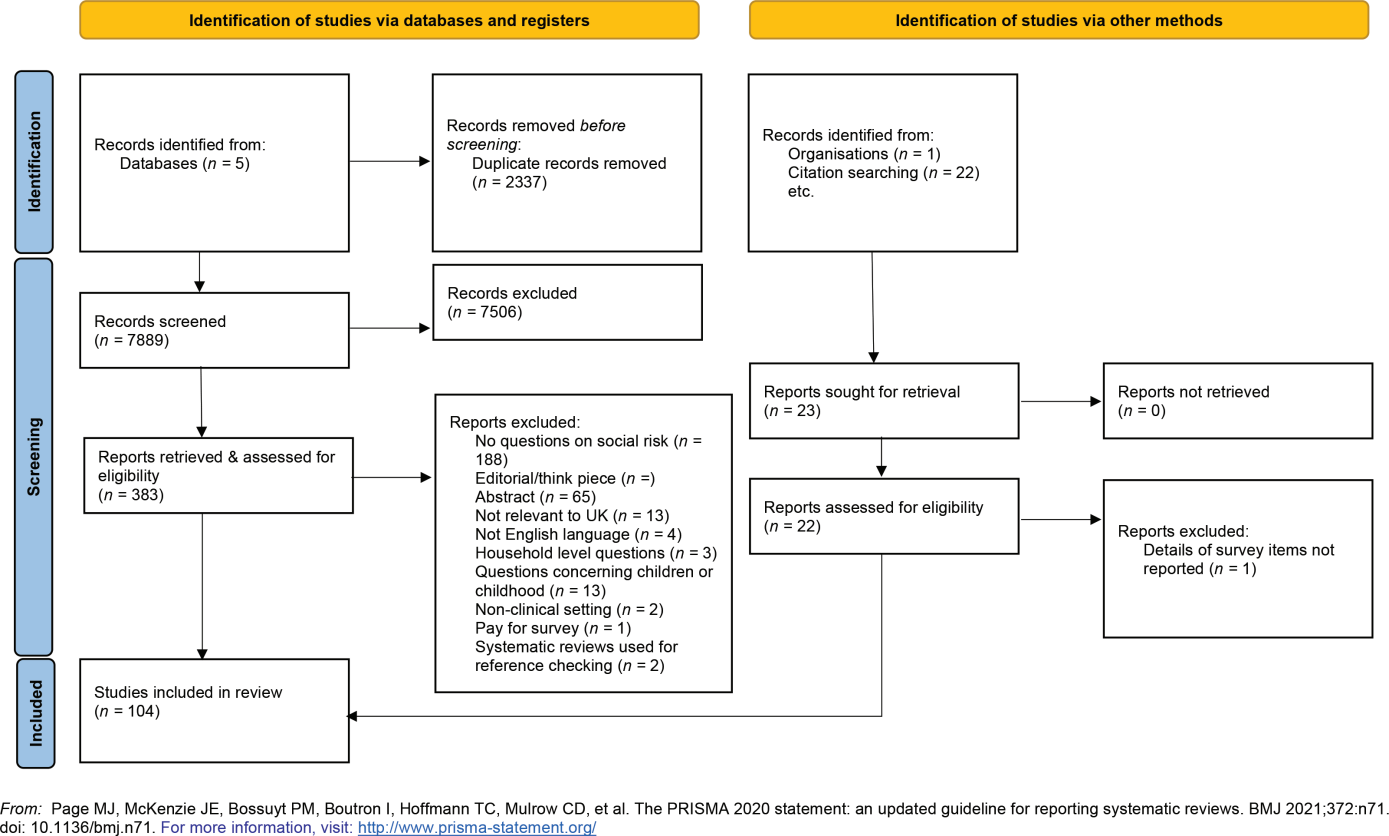
Preferred Reporting Items for Systematic reviews and Meta-Analyses (PRISMA) flow diagram. Preferred Reporting Items for Systematic reviews and Meta-Analyses (PRISMA) flow diagram. The full list of included studies^60–146^ is shown in Supplementary Tables S1–3

Searches identified 7889 unique citations. After full-text review and reference checking, 104 studies were included in the analysis (Figure 1). The majority of studies were based in the US (*n* = 82), followed by Canada (*n* = 9), multi-national (*n* = 3), UK (*n* = 3), Australia (*n* = 2), France (*n* = 2), New Zealand (*n* = 1), South Africa (*n* = 1), and Switzerland (*n* = 1) (Supplementary Table S3).

Tools and questions identified were used in the following settings: hospital-based specialties (*n* = 36), primary care (*n* = 26), defined populations (*n* = 16), community health centres (*n* = 9), emergency departments (ED) (*n* = 5), primary care and ED (*n* = 4), ED and specialty (*n* = 2), and school (*n* = 2) (Supplementary Table S3).

Screening commonly took place in clinical settings (*n* = 19), waiting rooms (*n* = 19), home (*n* = 17), before or after consultation (*n* = 12), during consultations (*n* = 9), and at check-in (*n* = 8). Written formats were the most frequently used (*n* = 34), along with face to face (*n* = 23), and electronic (*n* = 19) (Supplementary Table S3).

Duration to complete was reported inconsistently, but in the 15 studies where data were available times ranged from <1 minute to a median of 16 minutes (Supplementary Table S3).

Only two of the included studies reported all eight steps of the gold standard measurement in measure development^28,29^ (Supplementary Table S1).

The most commonly cited screening tools were the Accountable Health Communities Health-Related Social Needs Screening Tool, WE CARE, PRAPARE, and the Hunger Vital Sign (Supplementary Table S4, Supplement 3).

The most commonly cited longer screening tool was the US Department of Agriculture questionnaire (Supplement 3, Supplementary Table S5).

### Part 2: Delphi study

### Panel description

Of the panel members, 33% (*n* = 9) were aged between 45 years and 54 years, and 30% (*n* = 8) aged between 35 years and 44 years. The majority were female (74%) and of White ethnicity (56%). There were 30% Asian or Asian British, 7% Black or Afro-Caribbean, and 7% of mixed ethnicities (Supplementary Table S2). For survey 2 and 3 there were 22 and 21 responders, respectively.

#### Item reduction

**Figure 2.**
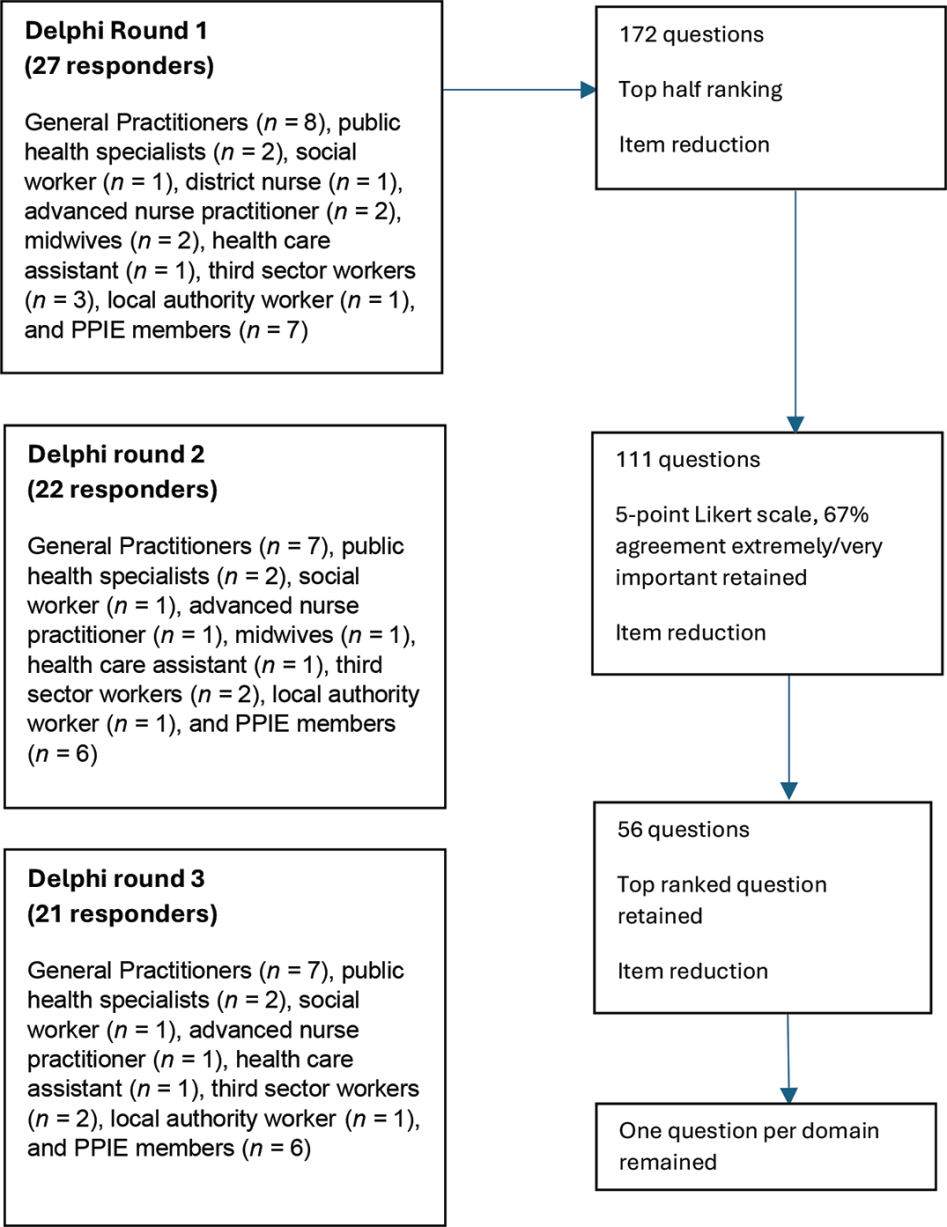
Delphi flow diagram. PPIE = patient and public involvement and engagement

The round 1 item reduction survey resulted in 111 of the 172 candidate items retained (Figure 2). Survey 2 led to 56 of the 111 items being retained (Supplementary Table S6). Of note at the end of this round, questions pertaining to an individual’s interest in further education scored low and were removed. The round 3 item reduction survey retained one item per each of the 10 domains (Supplementary Table S7), which resulted in the final screening tool (Table 2).

**Table 2.** Final 10-item primary care SDOH screening tool, with Delphi ratings of the top-rated screening question per domain from survey 3

Domain	Question with answer options	Reference	Mean	Median	Mean ranking
Financial situation	Do you have difficulty making ends meet at the end of the month? Always/Most of the time/Sometimes/Rarely	^35^	2.05	2	1
Food security	Do you worry that you would run out of food before you got money to buy more? Yes/No	^33^	3.43	2	1.67
Living conditions	Do you and your family have a safe and clean place to sleep? Yes/No	^32^	2.71	3	1.67
Housing situation	Do you have housing? Yes/No	^34^	3.14	3	1.67
Employment	What is your current work situation? Unemployed/Part-time or temporary work/Full-time work/Otherwise unemployed but not seeking work (ex-student, retired, disabled, unpaid primary care giver)/I choose not to answer this question	^36,37^	1.38	1	2
Utilities	Do you have trouble paying your heating or electricity bill? Yes/No	^38,39^	3.24	3	2.33
Social support	Do you have family and friends you can count on? Yes/No/Not sure	^31^	3.24	3	1
Neighbourhood safety	Do you feel safe in your neighbourhood? Yes/ No	^30^	1.24	1	1
Carer support	Do you have trouble taking care of a child, family member or friend? Yes/No	^38,39^	1.48	1	1
Transportation	Do problems with transport stop you from going to medical visits or getting medications? Yes/No	^33^	2.24	2	2

#### Mapping final screening tool items to gold standard tool measurement

The questions in the final SDOH screening tool (Table 2) were all generated by experts and seven had also been tested on a representative sample (Supplementary Table S1). Three questions met only one criterion;^30–32^ three met two criteria;^33,34^ one met three criteria;^35^ one met four criteria;^36,37^ and two met five criteria.^38,39^

Panel members ranked the domains they felt were most important to ask about with financial situation, food security, and living conditions being the top- ranked domains here (Supplementary Table S8).

#### Free-text comments

Categories that emerged from the data include: importance of screening question for understanding someone’s social risk; question structure and type; clarity of questions; temporal considerations (for example, whether someone’s social need is an ongoing problem, whether it is affecting them now or if it was a past issue); and methods for prioritising chosen questions. Questions that were direct with yes or no answers, specific questions that avoided vague terms such as 'having trouble', concerned the current situation, and those that had an immediate impact on health were preferred (Supplementary Table S9).

## Discussion

### Summary

We sought to identify screening tools and questions for collecting information on an individual’s SDOH for use in UK primary care. Most existing tools were US based, self-complete, conducted in clinical settings, and in written formats, with limited psychometric properties reported.

Using the Delphi process, we reached consensus among participants on key questions for assessing SDOH in UK primary care. Participants preferred direct, specific questions with binary responses, prioritising those addressing current situations and immediate health concerns.

### Strengths and limitations

The study’s limitations include reliance on published literature and restrictions to altering the questions in the Delphi study to not alter validity and reliability of the items. Important domains, such as domestic violence, health literacy, or asylum seeker status, were either not included as numerous screening tools already exist in the UK and to reduce responder burden. The sample size for the Delphi study was smaller than anticipated.

Strengths lie in our rigorous systematic review process, diverse stakeholder engagement, and PPIE involvement throughout.

### Comparison with existing literature

While other studies have synthesised SDOH screening tools and questions,^13,15,16,40^ our research updates this, emphasising questions that provide a UK context. Numerous social risk screening tools and questions were identified; however, few demonstrated the rigorous development required to meet all eight criteria of the gold standards tool, which questions their effectiveness for identifying social risk. These findings are consistent with previous studies^13,41^ and highlights the importance of ensuring any screening tool is validated to ensure they can identify those with unmet social need and who might benefit from intervention before widespread implementation. Responses to our Delphi survey highlighted that individuals favoured questions that were easy to understand, had binary response options, and asked about current problems that had an immediate impact on health. This is in comparison with previously reported perspectives on social risk screening tools, which included the need for it to be easy to perform and the importance of integrating screening into existing workflows.^42^

Our study found that screening mostly took place during clinical encounters; however, 17 studies reported screening took place at home, which is in contrast to other studies, primarily based in the US where screening rarely took place at home.^16,43^ Added to this, our review highlighted that the majority of screening was self-complete, which highlights a shift from previous findings where the majority were completed by someone else.^16^ Practically, self-complete screeners are likely to be less susceptible to responder bias and impact less on existing primary care workflows.

Understanding the benefits and costs of collecting individual SDOH data in a UK context is crucial. The growing literature on SDOH screening indicates a paradigm shift towards more formal and integrative screening for SDOH in clinical settings, suggesting UK policymakers should consider routine social risk screening in healthcare settings and follow examples set in the US, which has shown improved outcomes, including an increase in people receiving support, increased employment, reduced use of homelessness shelters, and improvement of children’s overall health status.^44,45^ Knowledge of people’s financial situation has been shown to lead to lower out-of-pocket costs.^46^ One of the few studies, by Singh *et al*, ^47^ which employed screening for SDOH in a UK specialist children’s and young people services setting, demonstrated that users were willing to complete the screening tool, that they welcomed being asked about social questions, and clinicians reported positively as they were able to focus on the issues most important to the service users.

### Implications for research and practice

Collecting SDOH data in healthcare settings has shown to improve health outcomes.^5^ A collaborative approach among public health, primary care, and government is essential to address unmet social need as typically resources required are beyond the scope of usual clinical care.^48^ Although the tool we propose has been developed primarily for use in clinical settings, the information collected could be used to inform NHS policy decisions and to underpin health inequalities research.

Key challenges to screening identified in the US include tools with uncertain validity and reliability, lack of training for clinicians, time constraints, and lack of acceptable and beneficial interventions when a need is identified.^49,50^ Arguably, screening without adequate resources could be counterproductive and lead to unintended harm.^51–53^ These obstacles and absence of national screening recommendation may account for limited adoption of screening in the UK.

Overall, perspectives on screening are positive and highlight the potential benefits.^10,54,55^ Examples of good practice can be seen in the US where people with unmet need are referred to local providers or signposted to available resources.^56^ Linking people to resources requires cross-sector collaboration, infrastructure funding, and leadership.

Screening for SDOH should be tailored for specific populations. The domains chosen reflect WHO and PROGRESS-Plus recommendations but different settings may prioritise different questions based on their unique challenges, for example, in a gynaecology setting domestic violence and unwanted pregnancy questions may be prioritised, and in primary care food and housing may be more important.^53^

Acceptability, workflow integration, and assessment of psychometric properties of the proposed screening tool need to be considered. In the US, targeted screening has helped to identify those most at risk,^57^ but may also introduce stigma and reinforcement of stereotypes.^58^

Using the SDOH screening tool will improve awareness of and address unmet need^59^ but further research is needed on its impact, potential unintended consequences, when screening should take place, who should undertake this and how.

In conclusion, numerous SDOH screening tools exist but few have been tested for psychometric properties or are applicable to UK settings. Our developed screening tool, informed by stakeholder input, is relevant to UK primary care. Future research should explore practicalities of screening, frequency, and how to address identified unmet need before routine implementation.

## Trial registration number

This study was registered in PROSPERO (CRD42023454870).

## Data

The dataset relied on in this article is available from the corresponding author on reasonable request.
